# *Commiphora myrrh* Supplementation Protects and Cures Ethanol-Induced Oxidative Alterations of Gastric Ulceration in Rats

**DOI:** 10.3390/antiox10111836

**Published:** 2021-11-19

**Authors:** Mohamed A. Lebda, Rabab E. Mostafa, Nabil M. Taha, Eman M. Abd El-Maksoud, Hossam G. Tohamy, Soad K. Al Jaouni, Ali H. El-Far, Mohamed S. Elfeky

**Affiliations:** 1 Department of Biochemistry, Faculty of Veterinary Medicine, Alexandria University, Alexandria 21526, Egypt; rabab.mostafa@alexu.edu.eg (R.E.M.); nabil.taha@alexu.edu.eg (N.M.T.); eman.abdelmaksoud@alexu.edu.eg (E.M.A.E.-M.); elfeky@alexu.edu.eg (M.S.E.); 2 Pathology Department, Faculty of Veterinary Medicine, Alexandria University, Alexandria 21526, Egypt; hossam.gafar@alexu.edu.eg; 3 Department of Hematology/Pediatric Oncology, Yousef Abdulatif Jameel Scientific Chair of Prophetic Medicine Application, Faculty of Medicine, King Abdulaziz University, Jeddah 21589, Saudi Arabia; saljaouni@kau.edu.sa; 4 Department of Biochemistry, Faculty of Veterinary Medicine, Damanhour University, Damanhour 22511, Egypt; ali.elfar@damanhour.edu.eg

**Keywords:** gastric ulcer, ethanol, myrrh, antioxidant

## Abstract

Gastric ulceration is a multifactorial disease defined as a defect in the gastric wall that extends through the muscularis mucosae into the deeper layers of the wall. The most common cause of gastric ulceration is alcohol consumption. In the current study, rats were gavaged by ethanol to investigate the protective (before ethanol) and curative (after ethanol) ability of *Commiphora myrrh* (myrrh) oil and extract against gastric ulcer oxidative alterations induced by ethanol. Myrrh significantly improved ulcer index, histomorphology, and periodic acid Schiff (PAS) impaired by ethanol. In addition, myrrh improved the antioxidant potential of gastric mucosa through enhancement of nuclear factor related to erythroid 2 (Nrf2), total glutathione (GSH), reduced GSH, and oxidized glutathione (GSSG), along with significant reduction in malondialdehyde (MDA) levels. Amelioration of gastric oxidative stress by myrrh enables gastric mucosa to counteract the ethanol’s inflammatory and apoptotic processes leading to improved gastric proliferation and healing. Interestingly, myrrh extract showed better protective and curative effects than myrrh oil against gastric ulceration.

## 1. Introduction

A peptic ulcer is defined as a lesion in the epithelium covering all of the digestive tract [1]. It interrupts the integrity of the gastrointestinal mucosal layer in the esophagus, stomach, and upper part of the small intestine [2]. A gastric ulcer is the most prevalent gastrointestinal disorder associated with an inflamed disruption in the mucous membrane layer of the stomach [3]. The annual frequency of this disease is globally about 8% [4]. Alcohol consumption has been linked to gastric mucosal inflammation, ulcers, and gastric cancer. [5].

The gastric ulcer has been developed by multiple endogenous aggressive factors such as high pepsin activity, bile reflux, abnormal gastric motility, minimum blood supply, hydrochloric acid (HCl) secretion, and *Helicobacter pylori* infection [6]. Furthermore, inappropriate eating habits [7], chemical agents as alcohol [8], stress [9], smoking [10], and prolonged excessive use of irritant drugs as non-steroidal anti-inflammatory drugs [11] are exogenous factors of gastric ulceration.

Ethanol is one of the primary causes of gastric ulcers and is used as a model for evaluating the gastroprotective effects of various drugs [12]. Ethanol is metabolized into acetaldehyde by the action of alcohol dehydrogenases; then, it is further metabolized to acetic acid, which has toxic effects on the stomach [13]. In addition, acetaldehyde was generated by a microsomal ethanol oxidizing system (cytochrome P450 2E1) and catalase [14].

Lessening gastric attacker mediators and promoting conservative gastric factors are the main therapeutic tools for restoring gastric slough [15]. The mechanism of gastric slough cure includes the reconditioning of the gastro-defensive ‘factors’ balance, creation of gastric mucosal cells, antioxidant status, and anti-inflammation effect [16].

*Commiphora myrrh* belongs to the family of Burseraceae and is grown in East Africa, Saudi Arabia, and India [17]. Many scientific studies have demonstrated the numerous benefits of using myrrh in medicine. These studies revealed that *Commiphora* species contained anti-inflammatory compounds [18], antinociceptive [19], immunostimulant [17], analgesic [18], and antioxidant [20] effects.

Few studies have been reported to investigate the potential effect of myrrh on the protection and treatment of gastric ulcers. Moreover, the exact mechanisms by which myrrh directly or indirectly affect gastric ulcers have not been elucidated. In addition, there is no clear report on the healing effect of myrrh on the gastric ulcer. It is required to conduct more detailed investigations to establish the anti-inflammatory, antioxidant properties, anti-apoptotic, and proliferating effect of myrrh in gastric ulcer treatment.

## 2. Materials and Methods

### 2.1. Ethics Statement

The study was approved by the Faculty of Veterinary Medicine Ethics Committee of Alexandria University, Egypt following the guidelines of “NIH Guide for the Care and Use of Laboratory Animals”.

### 2.2. Gas Chromatography-Mass Spectrometry (GC-MS) Analysis

The chemical compositions of samples were performed using a Trace GC-ISQ mass spectrometer (Thermo Scientific, Austin, TX, USA) following the method of described by Elsakhawy et al. [21].

### 2.3. Commiphora Myrrh

*C. myrrh* extract powder and myrrh oil were purchased from the local market in Souk El-Aser, Cairo, Egypt.

### 2.4. Animals, Housing, and Experimental Design

The study was carried on forty adult white male albino rats (150 ± 20 g) of two months of age. Rats were obtained from the Institute of Graduate Studies and Research, Alexandria University, Egypt. Animals were housed in separate cages and supplied with fresh and clean free water access. They were maintained on a balanced basal diet (Table S1). Rats were kept at constant environmental and nutritional conditions in a well-ventilated room under a 12/12-h light/dark cycle, optimum temperature (23 ± 2 °C), and humidity (55 ± 5%) throughout the experiment. The animals were left for two weeks for acclimatization before the beginning of the investigation.

Rats were randomly allocated into eight main groups; four of them were used for prevention experiment as the following:

Control: 5 rats received a basal diet with\free access to water without any treatment, and were sacrificed after 2 weeks.

EtOH: 5 rats were treated orally with absolute ethanol (99.8%) (Sigma–Aldrich, Louis, Mo, USA) solution of 5 mL/kg of B.W. and were then sacrificed after 1 h [22] of administration for induction of gastric ulcer.

MO-EtOH: 5 rats received a basal diet with free access to water, and were pretreated with myrrh oil MO in a dose of 400 mg/kg daily orally by gastric gavage for 2 weeks. Then rats received a single dose of ethanol (5 mL/kg B.W. orally). The rats were sacrificed after 1 h of ethanol administration.

ME-EtOH: 5 rats received a basal diet with free access to water, and were pretreated with myrrh extract ME in a dose of 400 mg/kg daily orally for 2 weeks, then received ethanol solution 5 mL/kg B.W orally as a single dose. The rats were sacrificed after 1 h of ethanol administration.

The other four groups were used for the treatment experiment of MO and ME after induction of gastric ulceration by ethanol as the followings:

Control: 5 rats received a basal diet with free access to water without any treatment and were sacrificed after 3 weeks as control.

EtOH: 5 rats were treated with ethanol solution of 5 mL/kg of B.W. and then received a basal diet with free access to water without any treatment for one week.

EtOH-MO: 5 rats were treated once with ethanol solution of 5 mL/kg of B.W. and then treated with myrrh oil MO in a dose of 400 mg/kg daily. It was administered orally by gastric gavage for one week. Then the rats were sacrificed.

EtOH-ME: 5 rats were treated once with ethanol solution of 5 mL/kg of B.W. and then treated with myrrh extract ME in a dose of 400 mg/kg daily. It was administered orally by gastric gavage for one week. Then the rats were sacrificed.

### 2.5. Sampling

The rats were fasted for 12 h and anesthetized using an intraperitoneal injection of (100 mg/kg of ketamine and 10 mg of xylazine per kg then euthanized, and the stomach samples were immediately dissected and rinsed with chilled normal saline 0.9%. Stomach samples were divided into three parts for biochemical analyses. The second part was maintained at −80 °C for RT-PCR assessment. The last part was cleansed with phosphate buffer saline (PBS, pH 7.4) and fixed in 4% paraformaldehyde (dissolved in PBS) for 48 h for sample fixation.

### 2.6. Assessment of Macroscopic Gastric Mucosal Injury

Stomach samples were opened along the greater curvature and rinsed with normal saline (NaCl 0.9%), and grossly examined for assessment for any lesions and imaged. The length of each lesion (mm) was measured according to Bozkurt et al. [23], and the gastric ulcer index (UI) was calculated following the method described by Das and Banerjee [24].

### 2.7. Histopathological Analysis

Fixed stomach specimens were processed and embedded in paraffin wax, cut at 4–5-µm thickness, and stained with H&E for light microscopy to assess the tissue sections for histopathological deflections and periodic acid Schiff (PAS) to detect mucosal glycoprotein [25].

### 2.8. Immunohistochemical Analysis and Quantitative Analysis

Immunohistochemical detection of proliferating cell nuclear antigen (PCNA), caspase-3, and tumor necrosis factor-alpha (TNF-α) was performed using the avidin-biotin-peroxidase technique as described by Mahmoud et al. [26]. The sections were then incubated for 1 h with the primary antibody specific for each PCNA (ab92552, Abcam, Cambridge, MA, USA), caspase-3 (9662, Cell Signaling Technology, Danvers, MA, USA), and TNF-α (ab6671, Abcam) diluted (1:500, 1:300, and 1:500, respectively) in PBS. After that, the sections were incubated with the appropriate biotinylated goat anti-rabbit secondary antibodies for one hour. Images of 10 different fields, at a magnification of (×400), were analyzed using ImageJ software to estimate positive brown immunostained cells’ area (%).

### 2.9. Biochemical Analyses

Parts of each rats’ stomach sample were homogenized in cold PBS and centrifuged for 10 min at 4 °C at 1435× *g*. Malondialdehyde (MDA), total glutathione (total GSH), reduced glutathione (GSH), oxidized glutathione (GSSG), and nitrite and nitrate (NOx) were determined by the commercial kits of Biodiagnostic Co. (Giza, Egypt). In addition, nuclear factor related to erythroid 2 (Nrf2) levels were determined using ELISA kits (Chongqing Biospes Co., Chongqing, China).

### 2.10. mRNA Extraction and Reverse Transcription-Polymerase Chain Reaction (RT-PCR)

Total RNA was extracted from the samples using easy-RED Total RNA Extraction Kits (iNtRON Biotechnology, Inc., Gyeonggi-do, Korea). The first-strand cDNA was generated using the HiSen Script cDNA (iNtRON Biotechnology, Inc.) package. Specific primers of α-SMA, iNOS, and TLR4 were used to amplify selected genes with glyceraldehyde 3-phosphate dehydrogenase (GAPDH) as a stable housekeeping gene (Table S2). Real-time Strata gene MX3005P PCR (Agilent Technologies, Santa Clara, CA, USA) and TOP real TM PreMIX SYBR Green qPCR master blend (cat. RT 500, Enzynomics, Daejeon, Korea) were used to determine mRNA expression. The relative gene expression levels were evaluated using the 2^−ΔΔct^ method described by Pfaffl [27].

### 2.11. Statistical Analysis

Data were statistically analyzed using general linear model’s procedures of SAS GLM (SAS, 2004). Percentage data were subjected to either the arcsine value or square roots according to the nature of the variable. Duncan’s multiple range tests were used for multiple comparison between means at (*p* < 0.05). Kolmogorov–Smirnov’s test was used to test the normal distribution of data (Duncan, 1955).

## 3. Results

### 3.1. Gas Chromatography-Mass Spectrometry (GC-MS) Analysis

Phytochemical components of myrrh oil and myrrh extract were detected with GC-MS analysis. Myrrh oil contained many ingredients such as 9-octadecenoic acid methyl ester (elaidic acid methyl ester) (43.52%), eugenol (11.96%), sitosterol (17.57), and thunbergol (11.32%) (Table 1).

Myrrh extract contains many compounds, including nizatidine (15.75%), 11-octadecenoic acid, methyl ester (14.68%), 9-octadecenoic Acid (Z)-, methyl ester (oleic acid) (14.68%), trans-13-octadecenoic acid (2.36%), hexadecanoic acid, and methyl ester (palmitic acid) (1.51%) (Table 2).

### 3.2. Macroscopic Gastric Mucosal Injury Investigation

**Figure 1:**
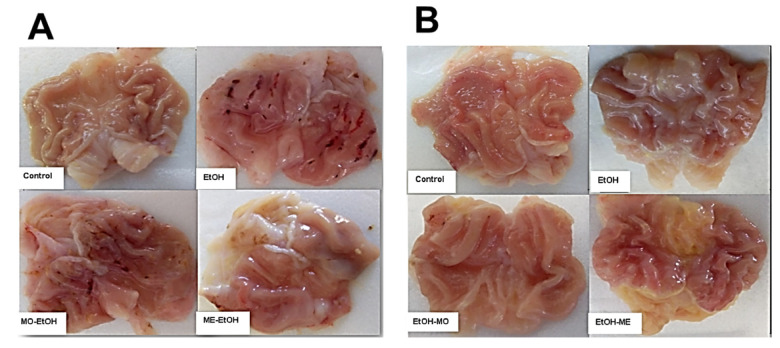
Gastric macroscopic lesions. (**A**) Preventive experiment: Control: showed the normal healthy pink color of gastric mucosa, normal gastric wall with normal mucosal thickening. No evident inflammation or ulcer. EtOH: stomach exhibited marked gross mucosal lesions, longitudinal hemorrhagic strikes of different sizes, and petechial lesions showed ulcerative inflammation associated with mucosal thinning. MO-EtOH showed mild hemorrhagic mucosal lesions and mild congestion. ME-EtOH; showed few congested mucosal lesions. (**B**) Treatment experiment: Control: showed healthy mucosa, normal folding, and normal thickening. No ulcer was recognized. EtOH: showed no mucosal lesions, moderate congestion, and hyperemia, with evidence of healing. EtOH-MO: showed a minute vascular response, no toxic or deteriorating effects with normal thickening as an evidence of healing. EtOH-MO: showed no hemorrhagic lesions with normal thickening as evidence of recovery.

Macroscopically, in prevention experimental groups, the control group showed the normal healthy pink color of gastric mucosa, normal folding, and normal mucosal thickening with no evident inflammation or ulcer. The gastric mucosa revealed severe congestion, hemorrhagic longitudinal irregular mucosal lesions of various diameters, and depth scattered across the entire gastric surface in the EtOH group. However, the preventive groups, MO-EtOH and ME-EtOH, significantly reduced the ethanol-induced gastric ulcer as preventive MO showed mild congestion, mild hemorrhagic mucosal lesions, and preventive ME showed few congested mucosal lesions (Figure 1A).

In addition, in the experimental treatment groups, the control group showed normal healthy gastric mucosa, normal folding, and normal mucosal thickening. The gastric mucosa and folding were improved with a moderate vascular response without hemorrhagic lesions in the EtOH group, while the MO-treated (EtOH-MO) group showed moderate congestion and hyperemia with normal thickening as evidence of healing. In addition, the ME-treated (EtOH-MO) group showed moderate vascular response (Figure 1B).

Ethanol administrations showed a significantly increased ulcer index compared with the control group. In comparison, prevention with MO and ME exerts significantly decreased gastric ulcer index compared with ethanol-treated animals. Moreover, there was no significant difference between prevention with MO and ME (Table 3).

Ethanol administrations showed a significantly increased ulcer index compared with the control group. In contrast, treatment with ME exerts significantly decreased gastric ulcer index compared with ethanol-treated groups. Moreover, there was no significant difference between treatment with MO and ME (Table 3).

### 3.3. Histological and Histochemical Assessment

**Figure 2:**
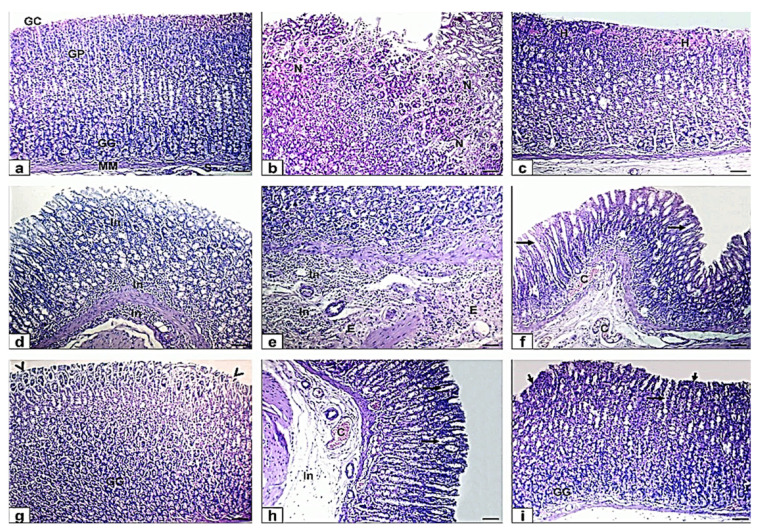
Photomicrographs of gastric sections stained with H&E in ethanol and ethanol-pretreated groups with myrrh extract (ME-EtOH)) and myrrh oil (MO-EtOH). (**a**) The normal control rats showed normal gastric mucosa, gastric cavity (GC), gastric pit (GP), gastric gland (GG), muscularis mucosa (MM), and submucosa (S). (**b**–**e**) Ethanol-induced ulcer in rats in one hour showing severe coagulative necrosis (N), hemorrhage (H), mononuclear cells infiltration, and aggregation (In) in all layers of gastric mucosa, and excessive edema (E) in the submucosa. (**f**,**g**) MO-pretreated rat showing dilated and wide gastric pits (long arrows), dilated congested blood vessels (C), and disruption to the surface epithelium (arrowheads). (**h**,**i**) ME-pretreated rat showing dilated gastric pits (long arrows), congested blood vessels (C) and intact appearance of histological structure of the epithelium (short arrows), gastric gland (GG), and mucosa layer. Scale bar= 100 µm.

Histopathological examination was executed to emphasize the morphological changes in the gastric tissues in the intended animal groups. The incidence and severity of lesions were summarized in Table 4. The stomach histology of control rats showed normal microscopic architecture with normal gastric mucosa, gastric cavity, squamous gastric epithelium, gastric pits, gastric glands, muscularis mucosa, and submucosa without any signs of abnormalities. In one hour, the gastric mucosa of ethanol-induced ulcer rats showed severe coagulative necrosis of entire mucosal thickness, hemorrhage, and intense mononuclear cells infiltration and aggregation in all layers of gastric mucosa beside excessive fait eosinophilic edema in submucosa, congested blood vessel, and disruption and destruction of the mucosa surface epithelium. Moreover, the microscopic findings in MO-EtOH-pretreated rats were dilated, and wide gastric pits dilated congested blood vessels and disorder the surface epithelium. Furthermore, ME-EtOH-pretreated rats exhibited wide gastric pits, congested blood vessels, and intact appearances of histological structure of the epithelium, gastric gland, and mucosa layer. The extravasation of RBCs in both pretreated rats was not detectable (Figure 2).

**Figure 3:**
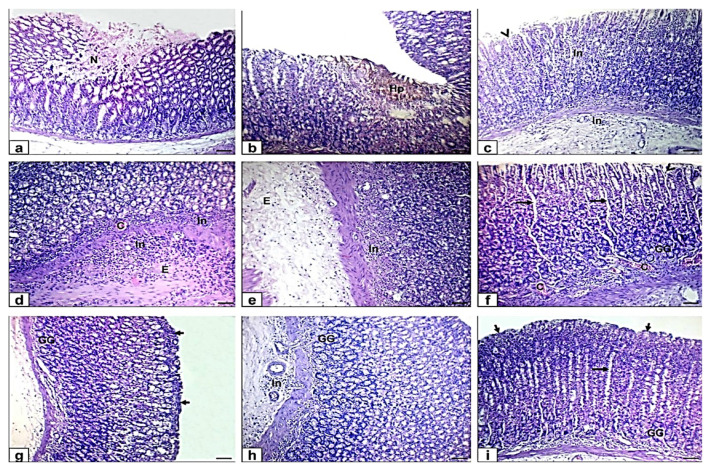
Photomicrographs of gastric sections stained with H&E in ethanol and ethanol-treated groups with myrrh extract (EtOH-ME) and myrrh oil (EtOH-MO). (**a**–**e**) Ethanol-induced ulcer in rats on day 7 showing moderate coagulative necrosis (N), old hemorrhages and brown hemosiderin precipitation (Hp), disruption to the surface epithelium (arrowhead), mononuclear cells infiltration and aggregation (In) in all layers of gastric mucosa, and moderate edema (E) in the submucosa. (**f**,**g**) MO treated rat showing dilated and wide gastric pits (long arrows), dilated congested blood vessels (C), and disruption to the surface epithelium (arrowhead). (**h**,**i**) ME-treated rat showing mild mononuclear cell infiltration (In), dilated gastric pits (long arrows), and intact appearance of histological structure of the epithelium (short arrows), gastric gland (GG) and mucosa layer. Scale bar = 100 µm.

The ethanol-induced ulcer rats on day 7 showed moderate coagulative necrosis, old hemorrhages with brown hemosiderin precipitation beside disruption to the surface epithelium, mononuclear cells aggregation in all layers of gastric mucosa, and mild edema in the submucosa. Moreover, EtOH-MO-treated rats showed wide gastric pits, blood vessels congestion, and disruption to the surface epithelium, while in EtOH-ME-treated rats, there were mild mononuclear cells infiltration, wide gastric pits, and intact appearance of histological structure of the epithelium and mucosa layer with no hemorrhages (Figure 3).

**Figure 4:**
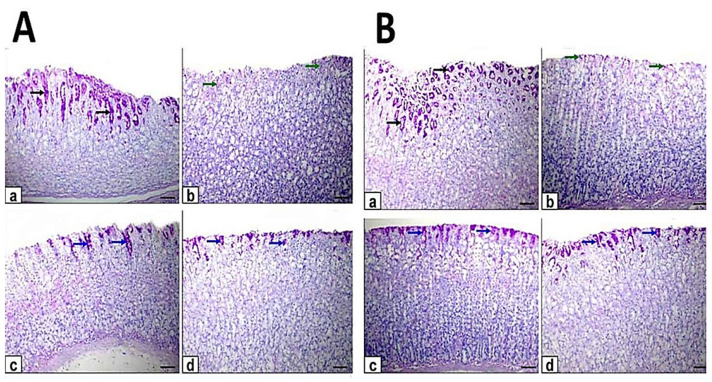
Photomicrographs of gastric sections stained with periodic acid Schiff (PAS). Scale bar = 100 µm. (**A**) The gastric sections of the pretreatment group: (**a**) control rats, (**b**) ethanol-induced ulcer rats on one hour, (**c**) MO-pretreated (MO-EtOH) rats, (**d**) ME-pretreated (ME-EtOH) rats. Arrows in the panels showed strong (black arrows), moderate (blue arrows), and faint (green arrows) PAS-positive reactions covering the surface epithelium. (**B**) The gastric sections of the treatment group: (**a**) control rats, (**b**) ethanol-induced ulcer rats on day 7, (**c**) MO-treated (EtOH-MO) rats, (**d**) ME-treated (EtOH-ME) rats. Arrows in the panels showed strong (black arrows), moderate (blue arrows), and faint (green arrows) PAS-positive reactions covering the surface epithelium.

Periodic acid Schiff histochemical staining was used to evaluate the status of glycoprotein production in the gastric epithelium, so the stomach sections of different groups were stained with PAS (Figure 4). The control rats’ specimens’ surface epithelial cells and the gastric glands showed strong magenta (reddish-purple) staining color of PAS reaction, indicating a thick and normal mucous layer. Stomach sections of ethanol-induced ulcer after one hour and 7 days showed an eroded mucosal layer with faint and weak staining color of PAS reaction. At the same time, pretreated rats exhibited a mild to moderate PAS reaction.

Rats in EtOH-MO and EtOH-ME showed moderate PAS reaction. The mean area percentage of the PAS-positive staining showed a significant decrease in ethanol-induced ulcers compared with the control. Moreover, myrrh oil and extract showed significant increased compared with ethanol-induced ulcer rats. This indicates that myrrh can protect against the glycoprotein content reduction induced by ethanol. The pretreated and treated rats with ME significantly increased area percentage of PAS-positive staining compared with MO (Table 5).

### 3.4. Immunohistochemical Assessment

**Figure 5:**
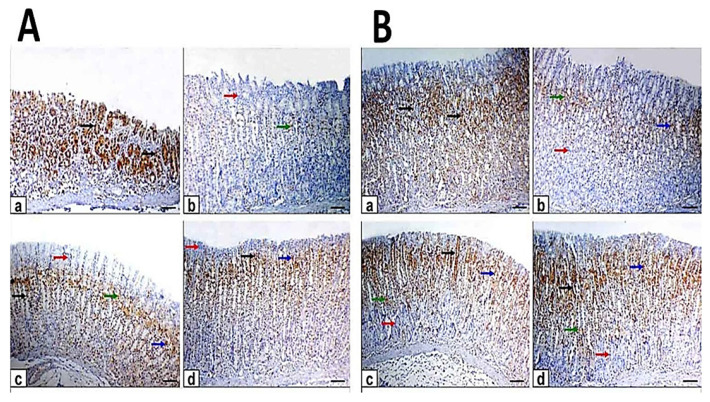
Photomicrographs of immunohistochemical staining of proliferating cell nuclear antigen (PCNA) protein expression in gastric sections. Scale bar = 100 µm. (**A**) The gastric sections of the pretreatment group: (**a**) control rats, (**b**) ethanol-induced ulcer rats on one hour, (**c**) MO-pretreated (MO-EtOH) rats, (**d**) ME-pretreated (ME-EtOH) rats. Arrows in the panels are showing strong (black arrows), moderate (blue arrows), weak (green arrows), and negative (red arrows) brown immunoreactions. (**B**) The gastric sections of the treatment group: (**a**) control rats, (**b**) ethanol-induced ulcer rats on day 7, (**c**) MO-treated (EtOH-MO) rats (**d**) ME treated (EtOH-ME) rats. Arrows in the panels showed strong (black arrows), moderate (blue arrows), weak (green arrows), and negative (red arrows) brown immunoreactions.

The gastric sections of control rats showed a stronger positive immunoreaction of PCNA protein expression as clarified by intense brown staining (Figure 5). In contrast, the ethanol-induced ulcer exhibited negative immunoreaction while in pretreated rats exhibited weak to moderate positive immunoreaction of PCNA, while in treated rats were showed moderate to strong positive immunoreactions of PCNA. The mean area percent of PCNA immunostaining positive cells was significantly decreased in ulcer compared with control. Moreover, the pretreated and treated rats with myrrh oil and extract showed significantly decreased compared with control and increased when compared with control and increased with ethanol-induced ulcer. In addition, the area % of PCNA positive cells in pretreated and treated rats with ME showed a significant increase when compared with MO (Table 3).

**Figure 6:**
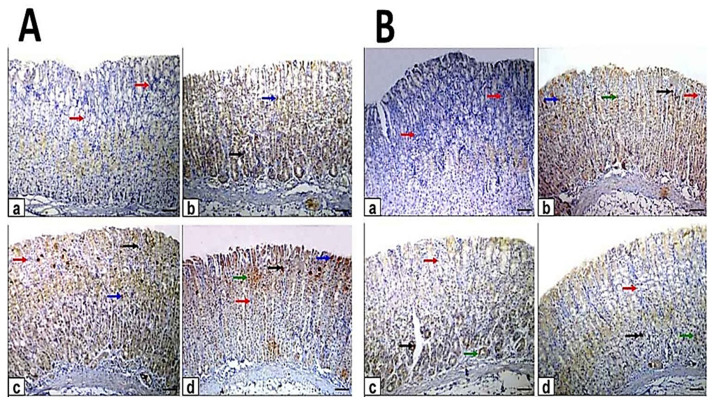
Photomicrographs of immunohistochemical staining of caspase-3 protein expression in gastric sections. Scale bar = 100 µm. (**A**) The gastric sections of the pretreatment group: (**a**) control rats, (**b**) ethanol-induced ulcer rats in one hour, (**c**) MO-pretreated (MO-EtOH) rats, (**d**) ME-pretreated (ME-EtOH) rats. Arrows in the panels are showing strong (black arrows), moderate (blue arrows), weak (green arrows), and negative (red arrows) brown immunoreactions. (**B**) The gastric sections of the treatment group: (**a**) control rats, (**b**) ethanol-induced ulcer rats on day 7, (**c**) MO-treated (EtOH-MO) rats, (**d**) ME-treated (EtOH-ME) rats. Arrows in the panels showed strong (black arrows), moderate (blue arrows), weak (green arrows), and negative (red arrows) brown immunoreactions.

Caspase-3 protein expression of the gastric section of the control group exhibited negative immunoreaction. Moreover, the ethanol induced ulcer displayed strong positive immunoreaction while in pretreated rats was moderate immunoreaction and in treated rats was weak to moderate positive immunoreaction of caspase-3 protein expression (Figure 6). The quantitative analysis of the area % of caspase-3 positive cells showed a significant increase in ethanol-induced ulcer compared with control. The pretreated and treated rats with myrrh oil and extract showed significant decreases compared with ethanol-induced ulcers and significant increases when compared with control. In addition, the area % of caspase-3 positive cells in pretreated and treated rats with ME showed a significant decrease when compared with MO (Table 3).

**Figure 7:**
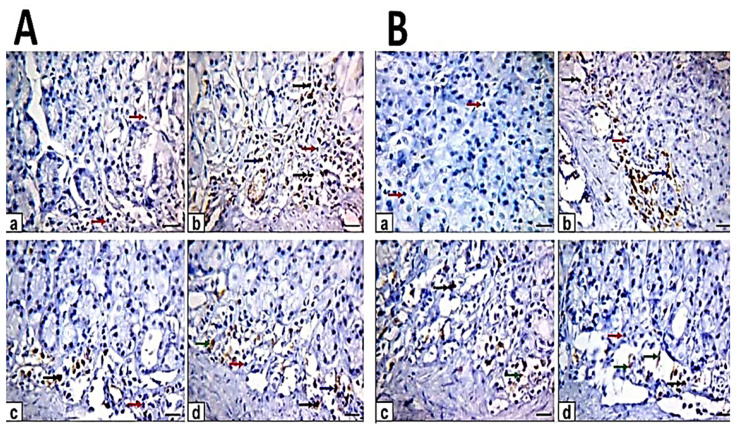
Photomicrographs of immunohistochemical staining of tumor necrosis factor-alpha (TNF-α) protein expression in gastric section. Scale bar = 100 µm. (**A**) The gastric sections of the pretreatment group: (**a**) control rats, (**b**) ethanol-induced ulcer rats in one hour, (**c**) MO-pretreated (MO-EtOH) rats, (**d**) ME-pretreated (ME-EtOH) rats. Arrows in the panels are showing strong (black arrows), moderate (blue arrows), weak (green arrows), and negative (red arrows) brown immunoreactions. (**B**) The gastric sections of the treatment group: (**a**) control rats, (**b**) ethanol-induced ulcer rats on day 7, (**c**) MO-treated (EtOH-MO) rats, (**d**) ME-treated (EtOH-ME) rats. Arrows in the panels showed strong (black arrows), moderate (blue arrows), weak (green arrows), and negative (red arrows) brown immunoreactions.

Immunohistochemical evaluation of gastric sections for TNF-α protein expression of control rats revealed minimal immunoreactivity. The ethanol-induced ulcer showed strong immunoreactivity while in pretreated rats there was moderate immunoreaction and in treated rats there was a weak to moderate positive immunoreaction of TNF-α protein expression (Figure 7). Quantitative analysis showed an increase in area (%) of TNF-α protein expression in ethanol-induced ulcers compared with control and other pretreated and treated rats. The pretreated and treated rats with myrrh oil and extract showed significantly decreased compared with ethanol-induced ulcers and increased when compared with control. In addition, the area % of TNF-α positive cells in pretreated and treated rats with ME showed a significant decrease when compared with MO (Table 3).

### 3.5. Biochemical Assessment

Compared with the control group, oral administrations of ethanol induced significant increases in gastric MDA and GSSG while exerting significant decreases in gastric reduced GSH, total GSH, and GSH/GSSG ratio. Although preventive MO and ME caused significant decreases in gastric MDA and GSSG, there were significant increases in reduced GSH, total GSH, and GSH/GSSG ratio compared with ethanol-treated animals. Moreover, rats with preventive ME produced a significantly increased total GSH and GSH/GSSG ratio and decreased MDA level compared with MO-EtOH (Table 6).

Ethanol administrations caused significant increases in gastric MDA and GSSG while exerting significant decreases in gastric reduced GSH, total GSH, and GSH/GSSG ratio compared with the control group, although treatment with MO and ME caused significant decreases in gastric MDA and GSSG and showed significant increases in the GSH/GSSG ratio compared with ethanol-treated animals. Moreover, there was no significant difference between rats treated with MO and ME (Table 6).

Ethanol administrations showed significant increases in gastric NOx and decreased gastric Nrf2 compared with the control group. However, prevention with MO and ME exerted significant decreases in gastric NOx and increased gastric Nrf2 compared with ethanol-treated animals. Moreover, there was no significant difference between rats pretreated with MO and ME (Table 7).

Ethanol administrations for one week showed significantly decreased gastric Nrf2 compared with the control group. At the same time, treatment with MO and ME exerted significantly increased gastric Nrf2 compared with ethanol-treated animals. Moreover, there were no significant differences between EtOH-MO and EtOH-ME treatment on gastric NOx (Table 5).

### 3.6. RT-PCR Assessment

Ethanol administrations showed significant increases in gastric iNOS, α-SMA, and TLR4 compared with the control group. In comparison with ethanol-treated rats, prevention with MO and ME exhibited significant decreases in gastric iNOS, α-SMA, and TLR4. Moreover, rats pretreated with MO exerted significant decreases gastric α-SMA compared with ME pretreated rats (Table 8).

Ethanol administrations showed significant increases in gastric iNOS, α-SMA, and TLR4 compared with the control group, while treatment with MO and ME significantly decreased gastric iNOS, α-SMA, and TLR4 compared with ethanol-treated animals. Moreover, rats pretreated with MO showed significantly decreased gastric α-SMA compared with ME pretreated animals.

## 4. Discussion

Gastric ulcers are still a global gastrointestinal disorder. Ethanol ingestion prompts intensified ulcer index, proven by marked gastric mucosal long hemorrhagic bands, petechial lesions with ulcerative inflammation [28], gastric degeneration and necrosis [29], death of epithelial cells, and local or multiple hemorrhagic ulcers [30]. This may be attributed to the high expression of various inflammatory cytokines and chemokine’s that are chemotactic to leukocytes and other cells of inflammatory processes [31].

Myrrh-treated groups exhibited a remarkable reduction of gastric lesions. Myrrh treatment was found to preserve the functional cytoarchitecture of the entire gastric mucosa, evidenced by a low ulcer index. In addition, myrrh implies improving the healing of the gastric ulcers and earlier preventing the development of ulcers induced by necrotizing agents [32]. Myrrh is a strong antimicrobial effect that inhibits the growth of different types of bacteria and fungi that hasten ulcer healing by myrrh [33].

Oxidative stress contributes to the pathogenesis of ethanol-induced gastric ulcers because the cytotoxicity of ethanol recruits inflammatory cells such as activated neutrophils and macrophages. These activated cells produced a rush of reactive oxygen species (ROS) that initiate gastric injury via lipid peroxidation and depletion of antioxidant defense [34]. ROS interacts with the cell membrane, causing lipid peroxidation, subsequently producing free radicals derived from highly reactive lipids such as MDA, causing oxidative gastric damage [35,36]. In the current study, ethanol-induced oxidative stress in this study via downregulation of Nrf2 gene expression, and its related gastric antioxidants system of reduced GSH, total GSH, and GSH/GSSG ratio, along with enhanced MDA levels. In the same context, ethanol-induced ulceration was associated with low Nrf2 expression, diminutions in gastric reduced GSH content, and enhanced MDA production [37,38,39].

Myrrh administration was hindered the oxidative stress by scavenging the free radicals mainly via the Nrf2 pathway. The study results relived that myrrh oil and extract exhibit upregulation of gastric Nrf2, reduced GSH, total GSH, and GSH/GSSG ratio with significant decreases in MDA levels. In addition, myrrh extract induced upregulation of Nrf2, and GSH along with diminished MDA levels [40,41,42]. The antioxidant potential of myrrh is due to the presence of thumbergol and eugenol with antioxidant power [33,43].

Inflammation is another major pathway that contributes to ethanol-induced gastric ulceration [44,45]. In the current study, the immunohistochemical area percentage of TNF-α, and enhanced gastric relative expression of TLR4 were significantly increased. TNF-α serves an important signaling role in the inflammatory cascade as it activates monocytes, macrophages, and gastric immune cells to produce PGE2, proteases, and chemotactic cytokines [46], suppress gastric microcirculation, cell proliferation, and angiogenesis at the ulcer margin, thus delays ulcer healing [47].

TLR4 is a potential inducer of the MAPK pathway, mediating the expression of numerous pro-inflammatory mediators and apoptotic alerts that engage in gastric mucosal damage [47]. In the current study, ethanol exposure for 1 h augmented gastric NOx and iNOS expressions. Excessive iNOS expression further consequent overproduction of NO is interrelated with tissue injury [48]. Ethanol causes immediate NO release through iNOS overexpression, which reacts with superoxide anion to form peroxynitrite (ONOO^−^), leads to lipid peroxidation, gastric damage, and accelerates gastric mucosal injury [49].

Interestingly, prevention with myrrh oil and extract exerts pronounced a decreased gastric NO level and iNOS expression compared with the ethanol-treated group in the early stage (prevention) but continued to increase the NO level in MO and ME treatment (late stage of inflammation). Active compounds from myrrh have been shown to increase iNOS expression [50]. Myrrh showed increased NO in rats with adjuvant-induced arthritis [51].

The gastric slough curative process encompasses various biological processes, including creating new blood vessels, reducing inflammation, and cell proliferation [52]. Neovascularization during ulcer healing is critical as the regeneration of blood microvessels is essential for oxygen and nutrient delivery to the healing site [53]. A detectable expression of PCNA was detected normally in gastric mucosa to evaluate the proliferative activity of gastric epithelial cells; it was strikingly diminished after ethanol administration [54]. Conversely, the obtained results proved that myrrh oil and extract exerted low α-SMA expression and an increased immunohistochemical area percentage of PCNA.

The apoptosis cascade has emerged as an important pathological pathway in the study of gastric ulcers, and it has been linked to oxidative stress and the inflammatory response. Intensive apoptosis of gastric epithelial cells induced by ethanol was due to enhanced caspase-3 activity [45]. Ethanol-induced apoptotic cell death of gastric mucosa was linked to excessive ROS and inflammation [55]. The current data revealed that myrrh oil and extract reduced the immunohistochemical area percentage of caspase-3. Eugenol in myrrh oil has anti-apoptotic action via suppression of caspase-3 [56]. The anti-apoptotic activity can be explained by myrrh’s ability to suppress ROS and TNF-α. Both have been reported to enhance gastric apoptosis.

Previous biochemical results were confirmed by histopathological assessment in which ethanol directly affected gastric mucosa and could directly affect gastric mucosa and induced necrosis and erosion of the surface gastric epithelial cells that agreed with Al Asmari et al. [57] and Lebda et al. [12]. Myrrh exhibited a protective effect on gastric mucosa as it promoted restoration and regeneration of gastric mucosa, diminished inflammatory cells infiltration, and cellular proliferation [58,59].

As shown in the PAS assessment, ethanol decreased the mean area percentage of PAS-positive staining score in mucosal cells due to the damaging effect of ethanol on the mucous cells and excessive oxidative stress [60]. On the contrary, myrrh induced an increment in the percentage of PAS-positive staining cells. Myrrh is known to increase mucus, nucleic acid production, and the concentration of non-protein sulfhydryl compound required for maintenance of gastroduodenal mucosa [32].

## 5. Conclusions

Injuries of the gastric mucosa are always associated with ethanol-induced gastric ulcers through enhancement of oxidative, inflammatory, and apoptotic processes. Myrrh supplementation before or after ethanol-induced gastric ulceration in rats significantly attenuated the oxidative alterations in gastric mucosa through increment of Nrf2 and gastric antioxidant potential, leading to improvement of gastric mucosal proliferation and injury healing. Therefore, myrrh is considered a promising preventive and curative feed supplementation in gastric ulceration.

## Figures and Tables

**Table 1:** Phytochemical analysis of myrrh oil.

Retention Time (Minute)	Phytochemical Compounds of Myrrh Oil	Area (%)
9.15	Eugenol	11.96
22.42	Elaidic acid, methyl ester	43.52
30.17	Sitosterol	17.57
32.18	Thunbergol	11.32

**Table 2:** Phytochemical analysis of myrrh extract.

Retention Time (Minute)	Phytochemical Compounds of Myrrh Extract	Area (%)
3.50	Diglycerol	10.24
4.52	Glycerin	16.09
9.17	Phenol, 2-methoxy-5-(1-Propenyl)-, (E)-	1.48
11.95	1-Dodecanamine,*N*,*N*-dimethyl	15.33
15.75	1-Tetradecanamine,*N*,*N*-dimethyl-	7.47
15.75	nizatidine	7.47
16.19	17-Octadecynoic acid	1.18
19.62	Hexadecanoic acid, methyl ester	1.51
22.40	Oleic acid	14.68
22.40	11-Octadecenoic acid, methyl ester	14.68
22.48	Trans-13-octadecenoic acid, methyl ester	2.36
22.57	2-Methylenebrexane	7.44
22.57	1-(Dimethylamino)-2-(benzy lamino)propane	7.44
22.81	Heptadecanoic acid, 16-methyl-, methyl ester	1.42
23.41	Ethyl oleate	0.99
24.42	9,12-Octadecadienal, dimethyl acetal	2.51
25.62	*N*-Methyl-*N*-benzyltetradecanamine	1.80

**Table 3:** Effect of myrrh oil and myrrh extract on ulcer index.

	Prevention				Treatment			
	Control	EtOH	MO-EtOH	ME-EtOH	Control	EtOH	EtOH-MO	EtOH-ME
Ulcer index	0.00 ± 0.00 ^c^	5.08 ± 0.18 ^a^	3.34 ± 0.19 ^b^	3.25 ± 0.22 ^b^	0.00 ± 0.00 ^c^	1.10 ± 0.17 ^a^	0.78 ± 0.15 ^ab^	0.60 ± 0.20 ^b^

Mean ± SE. Means denoted within the same row with different superscripts are significantly different (*p* < 0.05).

**Table 4:** Incidence and severity of histopathological lesions in the experimental groups.

	Ulcer								Prevention								Treatment							
	1 h				7 Days				MO-EtOH				ME-EtOH				EtOH-MO				EtOH-ME			
	−	+	++	+++	−	+	++	+++	−	+	++	+++	−	+	++	+++	−	+	++	+++	−	+	++	+++
Coagulative necrosis	0	1	1	3	0	2	2	1	2	2	1		3	1	1		3	2	0	0	3	2	0	0
Congestion of blood vessel	0	1	2	2	0	2	2	1	2	1	1	1	2	2	1	0	2	3	0	0	2	3	0	0
Hemorrhages	0	1	2	2	0	2	2	1	5	0	0	0	5	0	0	0	5	0	0	0	5	0	0	0
Edema	0	0	2	3	0	2	2	1	2	1	1	1	2	2	1	0	2	3	0	0	2	3	0	0
Inflammatory cell infiltration	0	1	1	3	0	1	3	1	2	1	2	0	2	2	1	0	2	2	1	0	2	3	0	0
Wide gastric pit	0	1	2	2	1	2	2	0	2	1	1	1	2	2	1	0	2	2	1	0	2	3	0	0
Ulcerative gastric surface	0	1	2	2	1	2	2	1	2	1	2	0	2	2	1	0	2	3	0	0	2	3	0	0
Intact gastric surface	5	0	0	0	3	2	0	0	0	1	3	1	0	1	2	2	0	1	2	2	0	0	2	3

Number of rats with lesions per total examined (5 rats per group). Severity of lesions was graded by estimating the percentage area affected in the entire section. Lesion scoring: (−) absence of the lesion = 0%, (+) mild = 5–25%, (++) moderate = 26–50%, and (+++) severe ≥50% of the examined tissue sections.

**Table 5:** Effect of myrrh oil and myrrh extract on periodic acid Schiff (PAS) staining and proliferating cell nuclear antigen (PCNA), caspase-3, and tumor necrosis factor-α (TNF-α) immunohistochemical staining.

	Prevention				Treatment			
	Control	EtOH	MO-EtOH	ME-EtOH	Control	EtOH	EtOH-MO	EtOH-ME
PAS (Area %)	31.20 ± 0.35 ^a^	5.82 ± 0.22 ^d^	11.64 ± 0.22 ^c^	15.94 ± 0.20 ^b^	31.20 ± 0.35 ^a^	18.52 ± 0.11 ^d^	22.92 ± 0.19 ^c^	26.18 ± 0.17 ^b^
PCNA (Area %)	17.74 ± 0.18 ^a^	3.74 ± 0.22 ^d^	8.36 ± 0.12 ^c^	9.72 ± 0.17 ^b^	17.74 ± 0.18 ^a^	6.72 ± 0.14 ^d^	11.92 ± 0.13 ^c^	14.18 ± 0.11 ^b^
Caspase-3 (Area %)	6.50 ± 0.30 ^d^	26.20 ± 0.30 ^a^	21.74 ± 0.26 ^b^	17.02 ± 0.13 ^c^	6.50 ± 0.30 ^d^	15.42 ± 0.14 ^a^	11.56 ± 0.23 ^b^	9.34 ± 0.21 ^c^
TNF-α (Area %)	0.46 ± 0.13 ^d^	15.16 ± 0.25 ^a^	12.34 ± 0.19 ^b^	9.54 ± 0.19 ^c^	0.46 ± 0.13 ^d^	9.50 ± 0.12 ^a^	7.76 ± 0.08 ^b^	5.86 ± 0.15 ^c^

Mean ± SE. Means denoted within the same row with different superscripts are significantly different (*p* < 0.05).

**Table 6:** Effect of myrrh oil and myrrh extract on malondialdehyde (MDA), total glutathione (total GSH), oxidized glutathione (GSSG), reduced GSH, and GSH/GSSG ratio.

	Prevention				Treatment			
	Control	EtOH	MO-EtOH	ME-EtOH	Control	EtOH	EtOH-MO	EtOH-ME
MDA (nmol/g tissue)	9.93 ± 0.73 ^c^	28.63 ± 1.03 ^a^	15.88 ± 0.41 ^b^	14.23 ± 0.49 ^b^	11.46 ± 0.32 ^b^	15.33 ± 0.68 ^a^	12.71 ± 0.39 ^b^	12.39 ± 0.25 ^b^
Total GSH (nmol/mg protein)	5.80 ± 0.18 ^a^	4.40 ± 0.08 ^c^	4.83 ± 0.18 ^bc^	5.20 ± 0.13 ^b^	5.60 ± 0.24 ^a^	4.73 ± 0.14 ^b^	4.99 ± 0.20 ^b^	5.15 ± 0.12 ^ab^
GSSG (nmol/mg protein)	0.38 ± 0.01 ^c^	0.66 ± 0.03 ^a^	0.49 ± 0.03 ^b^	0.44 ± 0.03 ^bc^	0.34 ± 0.01 ^b^	0.41 ± 0.01 ^a^	0.38 ± 0.02 ^b^	0.37 ± 0.02 ^b^
GSH (nmol/mg protein)	5.04 ± 0.15 ^a^	3.08 ± 0.02 ^d^	3.85 ± 0.13 ^c^	4.31 ± 0.12 ^b^	4.92 ± 0.22 ^a^	3.91 ± 0.12 ^b^	4.24 ± 0.18 ^b^	4.40 ± 0.09 ^b^
GSH/GSSG ratio	13.25 ± 0.10 ^a^	4.80 ± 0.21 ^d^	7.96 ± 0.24 ^c^	9.94 ± 0.83 ^b^	14.43 ± 0.21 ^a^	9.62 ± 0.04 ^c^	11.24 ± 0.50 ^b^	11.95 ± 0.75 ^b^

Mean ± SE. Means denoted within the same row with different superscripts are significantly different (*p* < 0.05).

**Table 7:** Effect of myrrh oil and myrrh extract on NOx and Nrf2.

	Prevention				Treatment			
	Control	EtOH	MO-EtOH	ME-EtOH	Control	EtOH	EtOH-MO	EtOH-ME
NOx (nmol/mg protein)	6.50 ± 0.23 ^b^	13.70 ± 0.64 ^a^	5.76 ± 0.39 ^b^	5.20 ± 0.46 ^b^	6.62 ± 0.18 ^a^	6.86 ±0.41 ^a^	6.14 ± 0.28 ^a^	6.00 ± 0.32 ^a^
Nrf2 (pg/mg protein)	125.88 ± 3.31 ^a^	67.21 ± 2.63 ^c^	109.50 ± 5.0 ^b^	106.24 ± 5.85 ^b^	124.36 ± 2.11 ^a^	96.70 ± 3.81 ^b^	117.06 ± 3.60 ^a^	120.96 ±1.47 ^a^

Mean ± SE. Means denoted within the same row with different superscripts are significantly different (*p* < 0.05).

**Table 8:** Effect of myrrh oil and myrrh extract on the relative expression of iNOS, α-SMA, and TLR4.

	Prevention				Treatment			
	Control	EtOH	MO-EtOH	ME-EtOH	Control	EtOH	EtOH-MO	EtOH-ME
iNOS	0.97 ± 0.09 ^c^	2.58 ± 0.12 ^a^	1.36 ± 0.06 ^b^	1.39 ± 0.05 ^b^	0.99 ± 0.09 ^c^	1.58 ± 0.06 ^a^	1.30 ± 0.14 ^b^	1.17 ± 0.04 ^bc^
α -SMA	0.99 ± 0.05 ^c^	3.17 ± 0.21 ^a^	1.09 ± 0.04 ^c^	1.47 ± 0.11 ^b^	1.04 ± 0.12 ^b^	1.52 ± 0.04 ^a^	1.11 ± 0.08 ^b^	1.04 ± 0.07 ^b^
TLR4	0.95 ± 0.16 ^b^	3.60 ± 0.31 ^a^	1.35 ± 0.10 ^b^	1.09 ± 0.07 ^b^	0.98 ± 0.15 ^b^	1.66 ± 0.21 ^a^	1.03 ± 0.08 ^b^	0.98 ± 0.08 ^b^

Mean ± SE. Means denoted within the same row with different superscripts are significantly different (*p* < 0.05).

## Data Availability

Data are contained within the article.

## Supplementary Materials

The following are available online at https://www.mdpi.com/article/10.3390/antiox10111836/s1, Table S1: Ingredients of basal diet, Table S2: Primer sequences of α-SMA, iNOS, TLR4, and GAPDH.
