# Supplementary material for: Commiphora myrrh Supplementation Protects and Cures Ethanol-Induced Oxidative Alterations of Gastric Ulceration in Rats
Source: Antioxidants (Basel). 2021 Nov 19;10(11):1836. doi: 10.3390/antiox10111836 (PMC8614819; doi:10.3390/antiox10111836)
Supplement: Supplementary file 1 [file antioxidants-10-01836-s001.zip › antioxidants-1397007-supplementary.pdf]

Table S1. Ingredients of basal diet.

**Ingredients**	**g/kg diet**
Corn flour	529.5
Casein	200
Sucrose	100
Soybean oil	70
Cellulose	50
Mineral mix	35
Vitamin mix	10
L-cystine	3
Choline	2.5

Table S2. Primer sequences of α-SMA, iNOS, TLR4, and GAPDH

Gene	Accession No.	Primer's sequence	
**α-SMA**	**NM_031004.2**	**F:**	5'- GAGCGTGGCTATTCCTTCGTG -3'
		**R:**	5'- CAGTGGCCATCTCATTTTCAAAGT -3'
**iNOS**	**NM_012611.3**	**F:**	5'-CACCACCCTCCTTGTTCAAC-3'
		**R:**	5'- CAATCCACAACTCGCTCCAA -3'
**TLR4**	**NM_019178.2**	**F:**	5'-AGCTTTGGTCAGTTGGCT-3'
		**R:**	5'-CAGGATGACACCATTGAAGC-3'
**GAPDH**	**NM_017008.4**	**F:**	5'-GGGTGTGAACCACGAGAAATA-3'
		**R:**	5'-AGTTGTCATGGATGACCTTGG-3'

GAPDH; glyceraldehyde 3-phosphate dehydrogenase, **iNOS**; inducible nitric oxide synthase, **TLR4**; Toll-like receptor 4, and **α-SMA**; α-smooth muscle actin.
